# 3D vs. 2D MRI radiomics in skeletal Ewing sarcoma: Feature reproducibility and preliminary machine learning analysis on neoadjuvant chemotherapy response prediction

**DOI:** 10.3389/fonc.2022.1016123

**Published:** 2022-12-02

**Authors:** Salvatore Gitto, Valentina D. A. Corino, Alessio Annovazzi, Estevāo Milazzo Machado, Marco Bologna, Lorenzo Marzorati, Domenico Albano, Carmelo Messina, Francesca Serpi, Vincenzo Anelli, Virginia Ferraresi, Carmine Zoccali, Alberto Aliprandi, Antonina Parafioriti, Alessandro Luzzati, Roberto Biagini, Luca Mainardi, Luca Maria Sconfienza

**Affiliations:** ^1^ Dipartimento di Scienze Biomediche per la Salute, Università degli Studi di Milano, Milan, Italy; ^2^ Department of Electronics, Information and Bioengineering (DEIB), Politecnico Di Milano, Milan, Italy; ^3^ Cardiotech Lab, Centro Cardiologico Monzino IRCCS, Milan, Italy; ^4^ Nuclear Medicine Unit, IRCCS Regina Elena National Cancer Institute, Rome, Italy; ^5^ International Medical School, Università degli Studi di Milano, Milan, Italy; ^6^ IRCCS Istituto Ortopedico Galeazzi, Milan, Italy; ^7^ Radiology and Diagnostic Imaging Unit, IRCCS Regina Elena National Cancer Institute, Rome, Italy; ^8^ Sarcomas and Rare Tumours Departmental Unit, IRCCS Regina Elena National Cancer Institute, Rome, Italy; ^9^ Department of Anatomical, Histological, Forensic and Musculoskeletal System Sciences, Sapienza University of Rome, Rome, Italy; ^10^ Oncological Orthopaedics Unit, IRCCS Regina Elena National Cancer Institute, Rome, Italy; ^11^ Istituti Clinici Zucchi, Monza, Italy; ^12^ Pathology Department, ASST Pini - CTO, Milan, Italy

**Keywords:** artificial intelligence, Ewing sarcoma, machine learning, magnetic resonance imaging, radiomics, texture analysis

## Abstract

**Objective:**

The extent of response to neoadjuvant chemotherapy predicts survival in Ewing sarcoma. This study focuses on MRI radiomics of skeletal Ewing sarcoma and aims to investigate feature reproducibility and machine learning prediction of response to neoadjuvant chemotherapy.

**Materials and methods:**

This retrospective study included thirty patients with biopsy-proven skeletal Ewing sarcoma, who were treated with neoadjuvant chemotherapy before surgery at two tertiary sarcoma centres. 7 patients were poor responders and 23 were good responders based on pathological assessment of the surgical specimen. On pre-treatment T1-weighted and T2-weighted MRI, 2D and 3D tumour segmentations were manually performed. Features were extracted from original and wavelet-transformed images. Feature reproducibility was assessed through small geometrical transformations of the regions of interest mimicking multiple manual delineations, and intraclass correlation coefficient >0.75 defined feature reproducibility. Feature selection also consisted of collinearity and significance analysis. After class balancing in the training cohort, three machine learning classifiers were trained and tested on unseen data using hold-out cross-validation.

**Results:**

1303 (77%) 3D and 620 (65%) 2D radiomic features were reproducible. 4 3D and 4 2D features passed feature selection. Logistic regression built upon 3D features achieved the best performance with 85% accuracy (AUC=0.9) in predicting response to neoadjuvant chemotherapy.

**Conclusion:**

Compared to 2D approach, 3D MRI radiomics of Ewing sarcoma had superior reproducibility and higher accuracy in predicting response to neoadjuvant chemotherapy, particularly when using logistic regression classifier.

## Introduction

Ewing sarcoma is the second most common bone sarcoma in children and adolescents after osteosarcoma (1). The standard of treatment in non-metastatic patients is represented by neoadjuvant chemotherapy, surgical resection and adjuvant chemotherapy (1). Radiotherapy can be administered to achieve local control post-operatively if surgical margins are inadequate or alone when complete resection is not feasible, particularly in certain anatomic locations (1). Five-year survival is 60-75% in localized disease, but it decreases substantially to < 20% in case of local or distant relapse (2). The extent of response to neoadjuvant chemotherapy predicts survival (3). Unfortunately, this can be assessed only after surgery based on histopathology (1). However, it would be desirable to know how the patient responded before surgery to tailor subsequent treatment accordingly. Furthermore, pathological response data are unavailable in non-operated patients treated with radiotherapy.

Radiomics includes extraction and analysis of quantitative parameters from medical images, known as radiomic features (4). Several features are routinely extracted in radiomic studies, but many of them are redundant or not informative for the clinical question of interest (5). Thus, a selection of radiomic features is necessary and feature reproducibility or stability assessment is an essential step of this process (5). However, a recent systematic review dealing with radiomics of musculoskeletal sarcomas highlighted that more than half of the papers lacked a preliminary analysis of feature reproducibility (6). In studies where this analysis was performed, intraclass correlation coefficient (ICC) was the most employed method to assess reproducibility (6). After selection, the optimal subset of radiomic features can be combined with machine learning algorithms for prediction purposes, including therapy response or outcome prediction (7, 8).

The aim of this study is twofold: (i) to assess the reproducibility of 3D and 2D radiomic features of skeletal Ewing sarcoma extracted from pre-treatment magnetic resonance imaging (MRI); and (ii) to preliminarily investigate 3D vs. 2D radiomics-based machine learning performance for prediction of response to neoadjuvant chemotherapy.

## Materials and methods

### Ethics

Institutional Review Board approved this retrospective study and waived the need for informed consent. All included patients granted written permission for anonymized data use for research purposes at the time of the MRI. After matching imaging, pathological and surgical data, our database was anonymized to delete any connections between data and patients’ identity according to the General Data Protection Regulation for Research Hospitals.

### Dataset description

This study was designed to meet the numerical requirements of reliability analyses in terms of patients involved, namely a minimum of 30, according to the ICC guidelines by Koo et al. (9). Thirty patients were retrospectively included at two tertiary bone sarcoma centres (centre 1, IRCCS Orthopaedic Institute Galeazzi, Milan, Italy; centre 2, IRCCS Regina Elena National Cancer Institute, Rome, Italy). Information was retrieved through medical records from the orthopaedic and pathology departments. Inclusion criteria were: (i) biopsy-proven Ewing sarcoma of the bone treated with neoadjuvant chemotherapy before surgery; (ii) response to neoadjuvant chemotherapy evaluated after surgery based on histopathology; (iii) 1.5- or 3-T MRI performed within two months before chemotherapy including turbo spin echo T1-weighted and T2-weighted sequences. Externally obtained MRI scans of patients referred to centres 1 and 2 were also included in this study as long as the minimal MRI protocol was available (MRI sequence parameters are detailed in **Supplementary Material**). Exclusion criteria were: (i) recurrent tumours; (ii) metastatic tumours; and (iii) image noise (motion or metal artifacts) affecting tumour segmentation. Neoadjuvant chemotherapy regimen consisted of 3-6 cycles including five to six drug combinations, namely doxorubicin, cyclophosphamide, ifosfamide, vincristine, dactinomycin and etoposide, as per current guidelines (1). Histopathologic response to neoadjuvant chemotherapy was assessed using the grading system by Picci et al. (also known as the “Bologna system”), where grade I (macroscopic residual tumour) defined poor response and grade II or III (microscopic or no residual disease, respectively) defined good response (10, 11). In our population of study, 7 patients were poor responders (all included at centre 1) and 23 were good responders (n=10 included at centre 1 and n=13 included at centre 2). Clinical data and demographics of the study population are detailed in **Table 1**.

**Table 1:** Clinical data and demographics of the study population.

Clinical data and demographics	
Age [median (1^st^-3^rd^ quartiles)]	18 (12-27) years
Sex	14 men; 16 women
Tumour location	Lower extremity: n=13
	Pelvis: n=7
	Spine: n=6
	Upper extremity: n=4
Histopathologic grading (10)	Grade I (poor response): n=7
	Grade II (good response): n=15
	Grade III (good response): n=8

Years of age are approximated to the nearest whole number.

### Segmentation

**Figure 1:**
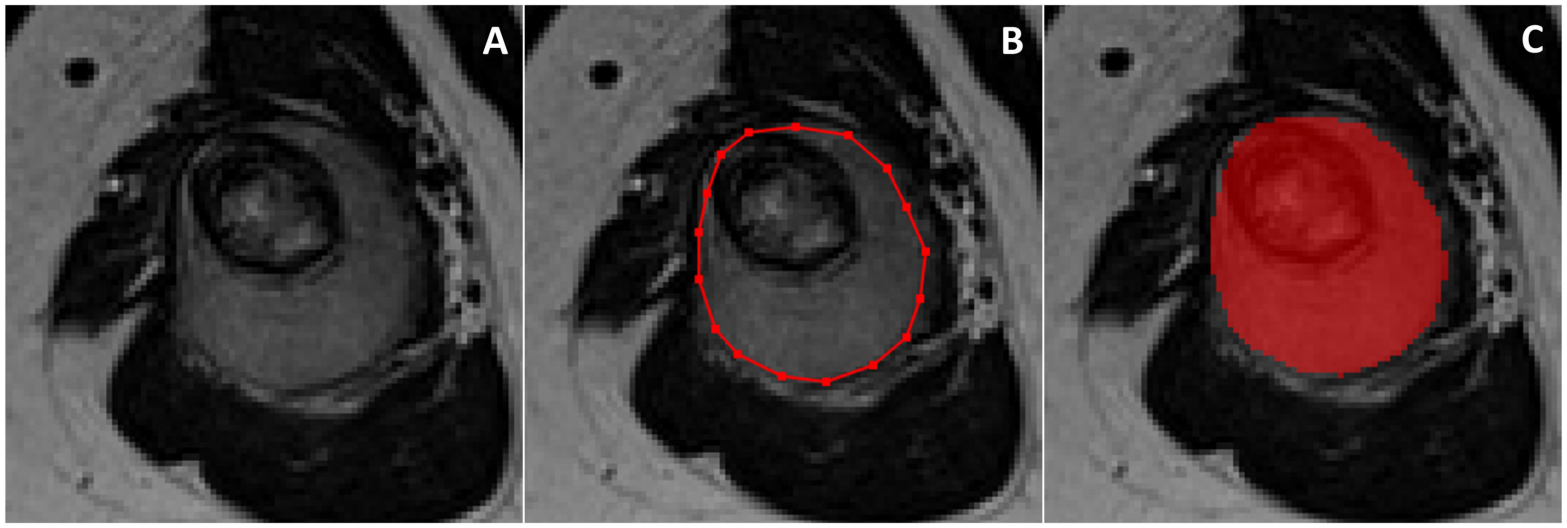
On the slice showing the largest tumor area **(A)**, 2D manual segmentation was obtained by drawing a polygonal region of interest **(B)** to include the whole tumor area **(C)**. The same procedure was repeated slice by slice to obtain 3D segmentation including the whole tumor volume.

All DICOM images were extracted and imported to the freely available, open-source software ITK-SNAP (v3.8) (12) for segmentation. On T1-weighted and T2-weighted MRI, manual segmentations were obtained by drawing both 2D regions of interest (ROIs) along tumour borders on the slice showing the largest tumour area and 3D ROIs including the whole tumour volume. The “polygon mode” ITK-SNAP tool was used for all segmentations (**Figure 1**). A musculoskeletal radiologist performed all segmentations. The radiologist knew the study would deal with Ewing sarcoma but was blinded to any information regarding disease course.

### Pre-processing and feature extraction

Image pre-processing and radiomic feature extraction were performed using PyRadiomics 3.0 (13). Five pre-processing steps were employed, including (i) grey-level normalization, (ii) pixel resampling and rescaling using cubic B-spline interpolation (resultant pixel size, 2x2x2 mm3), (iii) grey-level discretization (bin count, 32), (iv) denoising through the implementation of a 3D Gaussian filter, and (v) inhomogeneity correction using the open-source software 3D slicer (14). The default parameters of PyRadiomics were used, except for discretization. Particularly, intensity discretization with a fixed bin number was used instead of the fixed bin size discretization (default in PyRadiomics). The features extracted from each original image were grouped as follows (https://pyradiomics.readthedocs.io/en/latest/features.html): first-order features; shape-based features; grey-level cooccurrence matrix features; grey-level run length matrix features; grey-level size zone matrix features; grey-level dependence matrix features; neighbouring grey tone difference matrix features. First-order and textural features were extracted from both the original volumes and the 8 volumes obtained by first level wavelet decomposition (or 4 wavelet decompositions for 2D images) (15). For the full list of radiomic features, refer to PyRadiomics official documentation.

### Reproducibility analysis and feature selection

**Figure 2:**
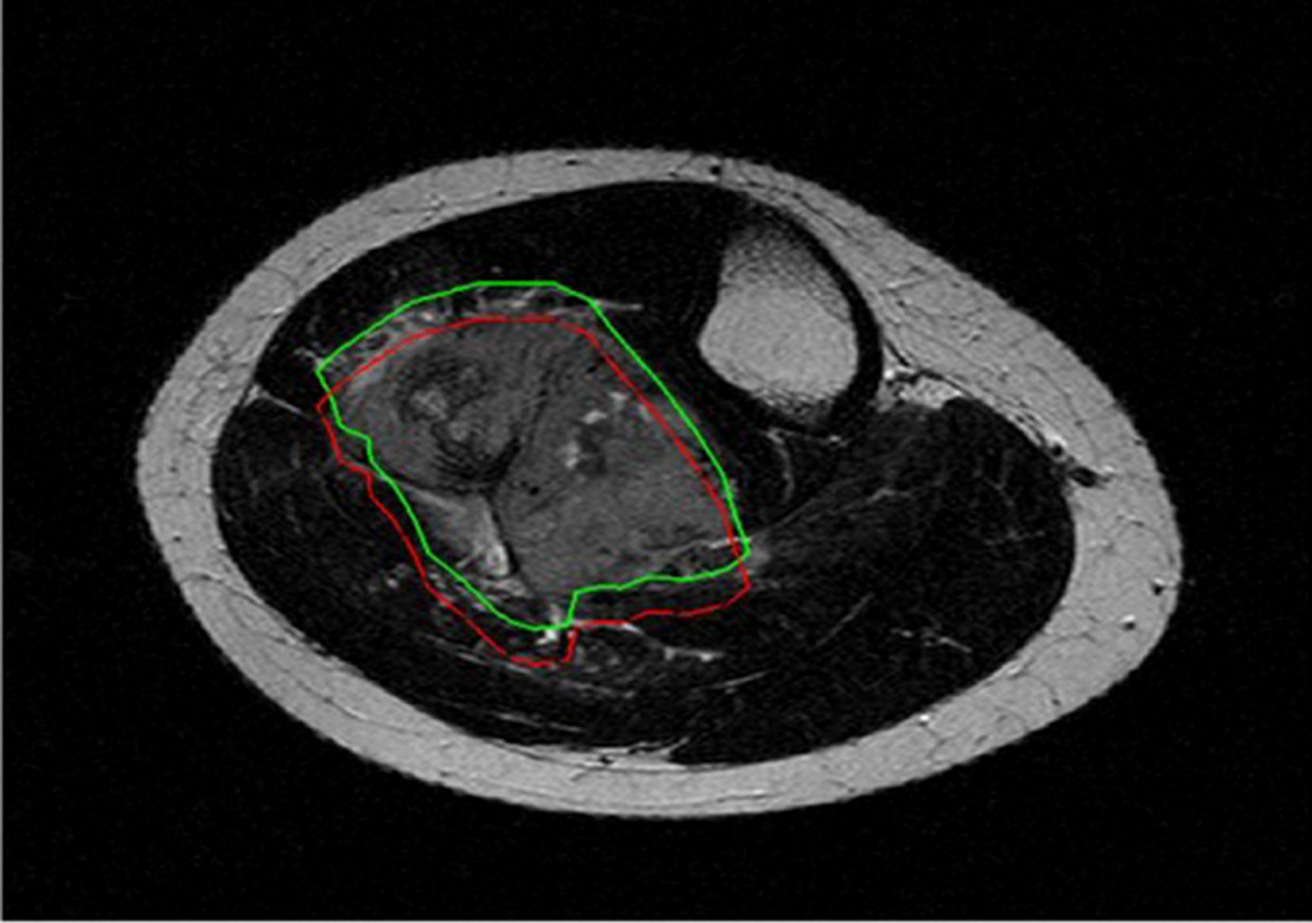
Original (green) and translated (red) version of 2D region of interest drawn around tumor borders on T2-weighted axial image.

Reproducibility analysis was performed to evaluate the robustness of radiomic features. Feature reproducibility was assessed through small geometrical transformations of the ROIs mimicking multiple manual delineations and the potential sources of intra- and inter-observer variability when multiple ROIs are drawn by the same or different readers, respectively (16, 17). Different translations of the ROI in the positive and negative direction of the x and y axes were applied. The entity of the translation was 10% of the length of the bounding box including the tumour (**Figure 2**). For each patient, radiomic features were extracted from five different ROIs, namely the original and 4 translated ROIs. ICC was used to quantify reproducibility, and radiomic features were considered stable if ICC > 0.75 (9). Among stable features, further dimensionality reduction was performed through collinearity and significance analysis. Collinearity was evaluated using Pearson correlation coefficient (r). The threshold for collinearity was set as r = 0.8. If a pair of features had high collinearity, the one with higher collinearity with others was excluded. Significance analysis addressed the ability of radiomic features to discriminate between good and poor responders. Wilcoxon rank-sum test was used. Radiomic features with statistically different distribution between these two groups (p-value < 0.05) were kept and ranked by their p-value.

### Machine learning-based classification

Based on the optimal subsets of radiomic features, classification models were built using three machine learning classifiers, namely k-nearest neighbours (k-NN, k = 1 linear nearest neighbour search with Euclidean distance function and no distance weighting), logistic regression (LR) and random forest (RF). To evaluate the unbiased performance of the classifiers, a cross-validation approach was used with a hold-out partition of 80-20 (80% for training and 20% for test sets, repeated 100 times). The same hold-out partition was used for 3D and 2D radiomics-based analysis. An imbalance between groups existed in our data (23 good vs. 7 poor responders), which could adversely affect classification performance. Therefore, prior to the training of the classifiers, class balancing was performed in the training cohort using the synthetic minority oversampling technique (18). This technique is used to artificially oversample the minority class, namely the poor responders in our study. After tuning on the training cohort, the classifiers were tested on unseen data, namely the test set.

### Statistical analysis

Continuous data are presented as median and interquartile (1^st^-3^rd^) range. Categorical data are presented as value counts and proportions. Differences in age and sex between good and poor responders were evaluated using Mann-Whitney U and chi-square tests, respectively. The reproducibility of 2D and 3D features was compared using t-test. The performances of the classifiers were evaluated and compared using area under the curve (AUC), accuracy, sensitivity, specificity, precision and balanced accuracy (i.e., the average between sensitivity and specificity) averaged on the test sets. Since LR and RF return continuous values, a binarization was necessary (the threshold for binarization was selected on the training set and then used on the test set). A two-tailed p-value < 0.05 was considered statistically significant. The statistical analysis was performed using MATLAB 2018b (Mathworks, Natick, MA, USA).

## Results

**Figure 3:**
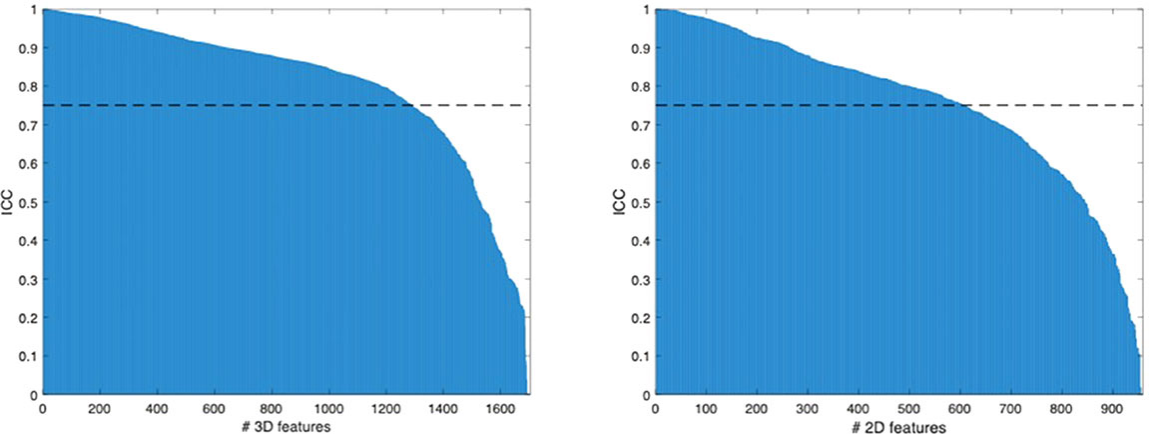
Radiomic features ranked by their ICC values.

No statistical difference in age (p = 0.841; 19 [12-29] vs. 18 [13-23] years) and sex (p = 0.526; 10 men and 13 women vs. 4 men and 3 women) was found between good and poor responders, respectively. A total of 1702 3D and 958 2D features were extracted per each patient. In detail, they were 851 3D and 479 2D features obtained from each MRI sequence. Among them, 1303 (77%) 3D and 620 (65%) 2D radiomic features were stable to geometrical transformations of the ROI (for 3D: 92% of the first order, 39% textural, 76% wavelet; for 2D: 89% of the first order, 33% textural, 61% wavelet), as shown in **Figure 3**.

After excluding features with high collinearity, the numbers of 3D and 2D features were further reduced to 101 and 58, respectively. Significance analysis yielded to four 3D and four 2D features, which were finally selected. In particular, the selected 3D features were all computed on the wavelet-transformed images and the selected 2D features were all obtained from the original images, respectively, as detailed in **Table 2**.

**Table 2:** List of selected features by feature class, ROI, MRI sequence and source image.

Selected feature	Feature class	ROI	Sequence	Source
Correlation	GLCM	3D	T1w	Wavelet
Minimum	First order	3D	T2w	Wavelet
Small Dependence High Gray Level Emphasis	GLDM	3D	T2w	Wavelet
Cluster Shade	GLCM	3D	T2w	Wavelet
Minimum	First order	2D	T1w	Original
Strength	NGTDM	2D	T1w	Original
Sphericity	Shape	2D	T2w	Original
Strength	NGTDM	2D	T2w	Original

GLCM, Gray Level Co-occurrence Matrix; GLDM, Gray Level Dependence Matrix; NGTDM, neighbouring grey tone difference matrix.

**Figure 4:**
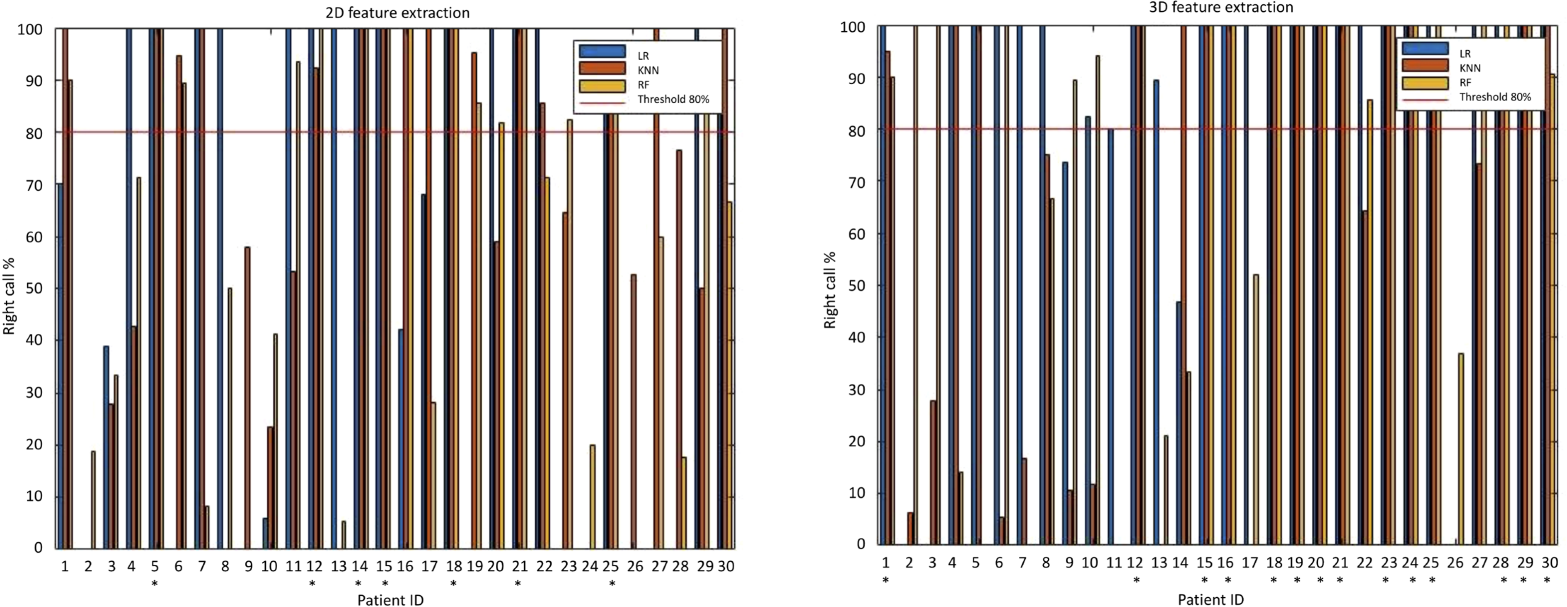
Column charts illustrating 2D and 3D radiomics-based machine learning classification performance. On the left, when using a percentage threshold of 80%, it is possible to note that only 7 patients were correctly classified with all classifiers in 2D analysis. On the right, an improvement was obtained in 3D analysis, where 14 patients were correctly classified at least 80% of times with all classifiers (correctly classified patients are marked with an asterisk). With 3D LR classifier (the best perforning one) 24 patients were correctly classified at least 80% of times.

The evaluation metrics for all classifiers are reported in **Table 3**. LR and RF classifiers achieved better accuracies when using 3D features compared to 2D features. Conversely, k-NN classifier showed better accuracy when built upon 2D features. As shown in column charts in **Figure 4**, only 7 patients were correctly classified with all 2D radiomics-based classifiers when using a percentage threshold of 80%. An improvement was obtained with 3D radiomics, as 14 patients were correctly classified at least 80% of times with all classifiers. LR built upon 3D features achieved the best performance with 85% sensitivity, 87% specificity and 85% accuracy (AUC=0.9) in predicting response to neoadjuvant chemotherapy, with 24 patients correctly classified at least 80% of the times.

**Table 3:** Evaluation metrics for LR, RF and k-NN classifiers using 2D and 3D radiomic features.

	3D - LR	3D - RF	3D – k-NN	2D - LR	2D - RF	2D – k-NN
Specificity	0.87 ± 0.34*	0.19 ± 0.39*	0.53 ± 0.50	1 ± 0.10	0.6 ± 0.49	0.54 ± 0.50
Sensitivity	0.85 ± 0.15*	0.92 ± 0.16*	0.66 ± 0.25*	0.47 ± 0.25	0.62 ± 0.24	0.74 ± 0.25
Balanced accuracy	0.86 ± 0.18*	0.55 ± 0.21	0.59 ± 0.25	0.73 ± 0.13	0.61 ± 0.26	0.64 ± 0.26
AUC	0.9 ± 0.13	0.72 ± 0.32	0.59 ± 0.25	0.86 ± 0.20	0.65 ± 0.32	0.64 ± 0.26
Accuracy	0.85 ± 0.13*	0.77 ± 0.14*	0.63 ± 0.20*	0.57 ± 0.20	0.62 ± 0.20	0.7 ± 0.21
Precision	0.97 ± 0.08*	0.82 ± 0.10*	0.87 ± 0.17	1 ± 0.03	0.88 ± 0.16	0.88 ± 0.14

*p < 0.05 comparison 2D vs. 3D.

## Discussion

This retrospective study compared the reproducibility of MRI radiomic features obtained from 2D and 3D segmentations and investigated radiomics-based machine learning prediction of response to neoadjuvant chemotherapy in Ewing sarcoma of the bone. 3D segmentations yielded higher rates of reproducible features compared to 2D approach. Additionally, LR built upon 3D features was the most accurate classifier in differentiating good from poor responders and achieved 85% accuracy (AUC = 0.9).

Despite its great potential to non-invasively quantify several tumour characteristics, radiomics still faces challenges preventing clinical transferability (8). A great variability in radiomic features has emerged as a major issue across studies, and segmentation is the most critical step (4). Therefore, in radiomic studies, preliminary methodological analyses are advisable to assess the robustness of different segmentation approaches and avoid biases due to non-reproducible, noisy features. This is in line with recent literature emphasizing the importance of reproducibility in artificial intelligence and radiology (19). Several strategies can be used to assess feature reproducibility, such as changes in image acquisition parameters (20) and multiple ROI delineations performed by different readers (21, 22), which are however time-consuming. In our study, feature reproducibility was evaluated through a time-saving method based on geometrical transformations of the ROIs mimicking multiple manual delineations (16, 17). In 3D and 2D segmentations, 77% and 65% MRI radiomic features were respectively stable to these transformations and then showed good overall reproducibility, although a certain degree of variability existed and highlighted the need for a preliminary reliability assessment.

With the introduction of neoadjuvant chemotherapy, the survival rates of Ewing sarcoma have substantially improved, although they are still low in patients with poor response (2). Accurate response evaluation using non-invasive approaches would be of paramount importance before surgery, as chemotherapeutic regimen can be adjusted and surgical options range from limb-sparing to radical surgery (1). Several imaging methods have been proposed to identify good and poor responders. Promising results have been obtained using diffusion MRI (23), dynamic contrast-enhanced MRI (24) or positron emission tomography-computed tomography (25). However, these methods often use a mean value to depict whole tumours, potentially overlooking tumour heterogeneity. Radiomics and machine learning have the potential to overcome these limitations (26–29), thus improving prognosis prediction. Our results showed that MRI radiomics-based machine learning could predict neoadjuvant chemotherapy response with up to 85% accuracy. Similar good performance has been described in previous studies dealing with neoadjuvant chemotherapy response prediction in osteosarcoma (30–32), where the superior predictive capacity of LR was also reported (30), as we observed with Ewing sarcoma. Based on our preliminary findings, we expect that radiomics-based machine learning may facilitate individualized assessment of neoadjuvant chemotherapy response and provide an effective tool for clinical decision-making.

Some limitations of this study need to be acknowledged. First, the design is retrospective, as a prospective analysis is not strictly necessary for radiomic studies (8). Second, the sample size was small. However, Ewing sarcoma of the bone is very rare, and our aim was first to evaluate the reproducibility of 3D and 2D MRI radiomic features. Consequently, our population met the minimum numerical requirements of a reliability analysis in terms of patients involved, according to ICC guidelines (9). Secondarily, machine learning prediction of neoadjuvant chemotherapy response was preliminarily explored. Third, good responders were over-represented compared to poor responders in our population of study. However, this reflected the incidence of good and poor response in our clinical practice, and class balancing was performed to artificially oversample the minority class in the training cohort (18). Fourth, the retrospective design accounts for the exclusion of contrast-enhanced MRI, which was not available in all our cases but helps in diagnosis of Ewing sarcoma (33) and deserves investigation in future radiomic studies. Fifth, a validation of the machine learning classifiers was not performed in an independent or external test cohort. The number of patients included at both our institutions was too small to be split into training and external test cohorts. Given the rarity of Ewing sarcoma, further multicentre investigations are warranted to involve more than two institutions, possibly located in different countries. This would allow for machine-learning model training, validation and independent testing on separate datasets, thus moving forward to clinical application.

In conclusion, 3D MRI radiomics of Ewing sarcoma had superior reproducibility and higher accuracy in predicting response to neoadjuvant chemotherapy, particularly when using LR classifier, compared to 2D approach. Although 3D segmentations are relatively time-consuming, they should be preferred in future studies needed to validate our machine learning model for therapy response prediction. If our preliminary findings are confirmed in a larger population, this method will allow for an individualized assessment of therapy response and subsequent treatment planning before surgery is performed, thus offering clinical decision support and having enormous potential for precision medicine.

## Data availability statement

The raw data supporting the conclusions of this article will be made available on request by the authors, without undue reservation.

## Ethics statement

Institutional Review Board approved this retrospective study and waived the need for informed consent.

## Author contributions

SG: Conceptualization, data curation, funding acquisition, investigation, methodology, project administration, visualization, writing – original draft. VC: Data curation, formal analysis, methodology, software, validation, writing – review and editing. AAn: Data curation, writing – review and editing. EM: Investigation, writing – review and editing. MB: Methodology, writing – review and editing. LoM: Methodology, writing – review and editing. DA: Investigation, writing – review and editing. CM: Investigation, writing – review and editing. FS: Investigation, writing – review and editing. VA: Resources, writing – review and editing. VF: Resources, writing – review and editing. CZ: Resources, writing – review and editing. AAl: Resources, writing – review and editing. AP: Resources, writing – review and editing. AL: Resources, writing – review and editing. RB: Resources, writing – review and editing. LuM: Methodology, supervision, writing – review and editing. LS: Conceptualization, funding acquisition, project administration, resources, supervision, writing – review and editing. All authors read and approved the final version of the manuscript. All authors contributed to the article and approved the submitted version.

## Funding

This research was supported by the Post-Doctoral Fellowship awarded by Fondazione Umberto Veronesi (SG) and Investigator Grant awarded by Fondazione AIRC per la Ricerca sul Cancro for the project "RADIOmics-based machine-learning classification of BOne and Soft Tissue Tumors (RADIO-BOSTT)" (LS). The funding sources provided financial support without any influence on the study design; on the collection, analysis, and interpretation of data; and on the writing of the report. The first and last authors had the final responsibility for the decision to submit the paper for publication.

## Conflict of interest

The authors declare that the research was conducted in the absence of any commercial or financial relationships that could be construed as a potential conflict of interest.

## Publisher’s note

All claims expressed in this article are solely those of the authors and do not necessarily represent those of their affiliated organizations, or those of the publisher, the editors and the reviewers. Any product that may be evaluated in this article, or claim that may be made by its manufacturer, is not guaranteed or endorsed by the publisher.

## Abbreviations

AUC, area under the curve; ICC, intraclass correlation coefficient; k-NN, k-nearest neighbours; LR, logistic regression; MRI, magnetic resonance imaging; RF, random forest; ROI, region of interest.
