# Supplementary material for: 3D vs. 2D MRI radiomics in skeletal Ewing sarcoma: Feature reproducibility and preliminary machine learning analysis on neoadjuvant chemotherapy response prediction
Source: Front Oncol. 2022 Dec 2;12:1016123. doi: 10.3389/fonc.2022.1016123 (PMC9755864; doi:10.3389/fonc.2022.1016123)
Supplement: Supplementary file 1 [file DataSheet_1.pdf]

3D vs. 2D MRI radiomics in skeletal Ewing sarcoma: Feature reproducibility and preliminary machine learning analysis on neoadjuvant chemotherapy response prediction

Salvatore Gitto; Valentina D.A. Corino; Alessio Annovazzi; Estevão Milazzo Machado; Marco Bologna; Lorenzo Marzorati; Domenico Albano; Carmelo Messina; Francesca Serpi; Vincenzo Anelli; Virginia Ferraresi; Carmine Zoccali; Alberto Aliprandi; Antonina Parafioriti; Alessandro Luzzati; Roberto Biagini; Luca Mainardi; Luca Maria Sconfienza

SUPPLEMENTARY MATERIAL

Supplementary table. MRI acquisition parameters for each patient and sequence.

Pt. ID	Location	T1w MRI			T2w MRI		
		Plane	Slice thickness (mm)	Matrix	Plane	Slice thickness (mm)	Matrix
1	upper extremity	axial	3.5	320x320	axial	3.5	320x320
2	spine	sagittal	4	384x378	axial	5	448x408
3	pelvis	axial	4	448x448	axial	4	448x448
4	upper extremity	axial	4	320x320	axial	4	320x320
5	lower extremity	coronal	3.5	512x512	axial	3	560x560
6	lower extremity	axial	6	320x320	axial	6	320x320
7	lower extremity	axial	3.5	320x256	sagittal	3	320x320
8	pelvis	axial	3.5	448x348	axial	3	448x348
9	pelvis	axial	4.5	864x864	axial	4.5	864x864
10	spine	sagittal	4	640x640	sagittal	4	640x640
11	spine	axial	3.5	512x512	axial	3.5	512x512
12	lower extremity	coronal	3	448x448	axial	5	448x348
13	lower extremity	axial	3.5	320x320	axial	3.5	320x320
14	spine	axial	4	400x400	axial	4	400x400
15	lower extremity	axial	5	288x288	axial	5	288x288
16	spine	axial	3	288x288	axial	4	288x288
17	pelvis	coronal	3.5	384x384	axial	3.5	320x320
18	lower extremity	axial	5	256x256	axial	5	256x256
19	lower extremity	axial	5	256x256	axial	5	256x256
20	lower extremity	axial	8	256x256	axial	6	256x256
21	spine	axial	4	512x512	sagittal	4	512x512
22	lower extremity	axial	4	512x512	axial	4	512x512
23	pelvis	axial	5	512x512	axial	5	512x512
24	upper extremity	coronal	4	512x512	coronal	4	512x512
25	lower extremity	sagittal	3	320x320	axial	3.5	384x384
26	pelvis	axial	5	512x512	axial	5	512x512
27	lower extremity	axial	4.5	864x864	axial	4.5	864x864
28	lower extremity	sagittal	4	512x512	axial	4	512x512
29	upper extremity	axial	4	320x320	coronal	4	320x320
30	pelvis	axial	5.5	438x336	axial	5.5	438x336

Supplementary statistical analysis

As different MRI scanners were used, we performed statistical tests to assess the presence of any batch effect due to the different scanners.

Supplementary Figure A shows the histogram of the various scanners present in the dataset.

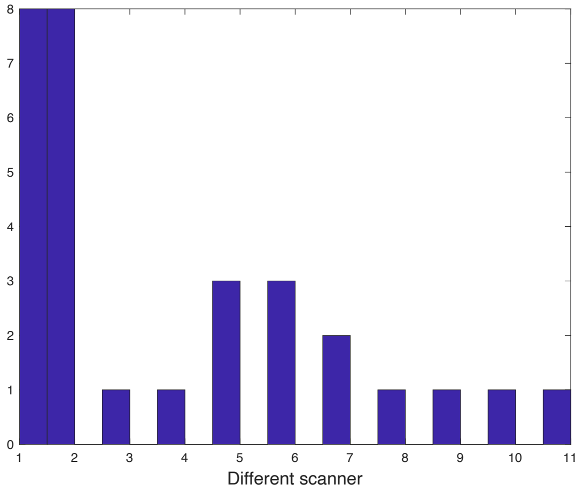

Supplementary Figure A. Different scanners in the dataset.

We performed Principal Component Analysis (PCA). We plotted the data in a space of reduced dimensions (Supplementary Figure B) and by visual inspection we observed no batch effect.

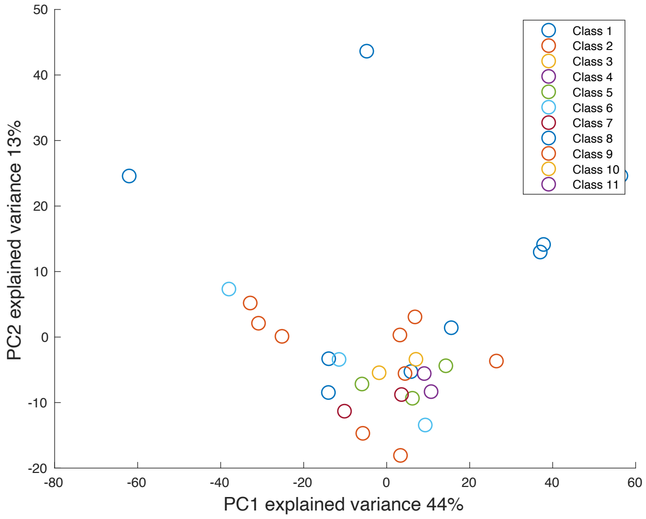

Supplementary Figure B. PCA results, PC 1: First Principal Component, PC 2: Second Principal Component, EV: Explained Variance.

We also used t-distributed stochastic neighbor embedding (tSNE) to check for batch effect using the Euclidean distance. As shown in Supplementary Figure C, our results suggested the absence of clusters.

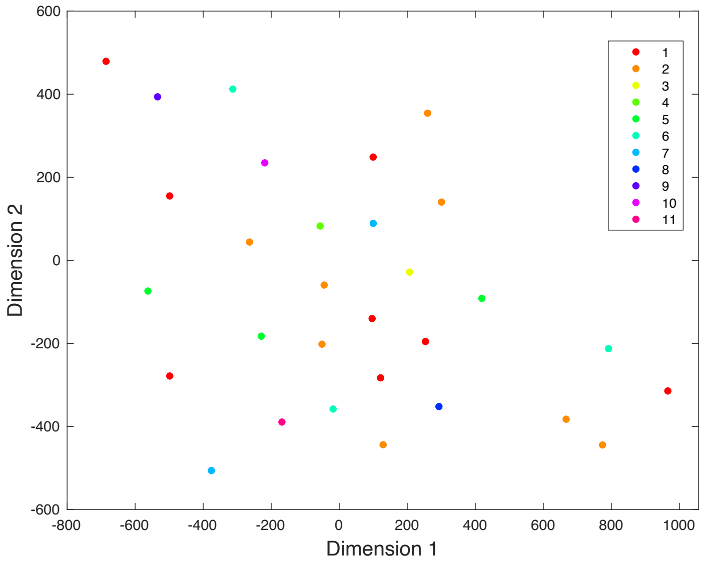

Supplementary Figure C. t-SNE results for the Euclidean distance.

Finally, we performed the Kruskal–Wallis test on both the first and second main component scores and the absence of clusters was confirmed, being the p-value 0.78 and 0.36 for the first and second principal component, respectively.

As six scanners were used for one patient each, we reran all the above-described analyses considering only the other five scanners that were used for more than one patient. Also with this modification, our results suggested that no batch effect was present in our data.
